# Supplementary material for: The mutational landscape of human olfactory G protein-coupled receptors
Source: BMC Biol. 2021 Feb 5;19:21. doi: 10.1186/s12915-021-00962-0 (PMC7866472; doi:10.1186/s12915-021-00962-0)
Supplement: Supplementary file 1 — Additional file 1: Table S1. Nucleotide sequencing data sources used in the study. Table S2. Number of mutations in human ORs collected in the study. Table S3. Human ORs genes with functional annotation excluded from the study. Table S4. Conserved topological sites with functional implication in the GPCR activity. Table S5. Non-olfactory class A GPCRs used in topological annotation. [file 12915_2021_962_MOESM1_ESM.docx]

**Additional file 1**

**The mutational landscape of human olfactory G protein-coupled receptors**

Ramón Cierco Jimenez ^1^, Nil Casajuana-Martin ^1^, Adrián García-Recio ^1^, Lidia Alcántara ^1^, Leonardo Pardo ^1^, Mercedes Campillo ^1^ and Angel Gonzalez ^1,^*

^1^ Laboratori de Medicina Computacional, Unitat de Bioestadística, Facultat de Medicina, Universitat Autònoma de Barcelona, E-08193 Bellaterra, Spain.

Present Address: Ramón Cierco Jiménez, International Agency for Research on Cancer, Evidence Synthesis and Classification Section, WHO Classification of Tumours Group, 150 Cours Albert Thomas, 69008 Lyon, France.

* To whom correspondence should be addressed.

Email: Angel.Gonzalez@uab.es

**Table S1: Nucleotide sequencing data sources used in the study.** Human OR mutation data was taken from the genome aggregation database (gnomAD v2), comprising a total of 16 million single nucleotide variations (SNVs) and 1.2 million indels from 125.748 exomes, and 229 million SNVs and 33 million indels from 15.708 genomes. Samples were subdivided into seven geographic ancestries according to a random forest classifier using principal component analysis (PCA), plus an eighth group named “Other” (OTH) that include individuals that do not unambiguously cluster within any of the foregoing populations.

| **Population** | **Abbr.** | **Genomes** | **Exomes** | **Total** |
| --- | --- | --- | --- | --- |
| African/African-American | AFR | 4,359 | 8,128 | 12,487 |
| Admixed American (Latino) | LAT | 419 | 16,791 | 17,210 |
| Ashkenazi Jewish | ASH | 145 | 5,040 | 5,185 |
| East Asian | EA | 780 | 9,197 | 9,977 |
| European Finnish | EF | 1,738 | 10,824 | 12,562 |
| European Non-Finnish | ENF | 7,718 | 56,885 | 64,603 |
| South Asian | SA | 0 | 15,308 | 15,308 |
| Other (population not assigned) | OTH | 544 | 3,070 | 3,614 |
| **Total** |  | **15,708** | **125,748** | **141,456** |

**Table S2: Number of mutations in human ORs collected in the study.** The table shows the number of nucleotide variants identified in 378 functional OR genes belonging to 17 OR families.

| **OR Family 1** | | **OR Family 2** | | **OR Family 3** | | **OR Family 4** | | **OR Family 5** | | **OR Family 6** | | **OR Family 7** | | **OR Family 8** | | **OR Family 9** | | **OR Family 10** | | **OR Family 11** | | **OR Family 12** | | **OR Family 13** | | **OR Family 14** | | **OR Family 51** | | **OR Family 52** | | **OR Family 56** | |
| --- | --- | --- | --- | --- | --- | --- | --- | --- | --- | --- | --- | --- | --- | --- | --- | --- | --- | --- | --- | --- | --- | --- | --- | --- | --- | --- | --- | --- | --- | --- | --- | --- | --- |
| OR  Name | №  Mut | OR  Name | №  Mut | OR  Name | №  Mut | OR  Name | №  Mut | OR  Name | №  Mut | OR  Name | №  Mut | OR  Name | №  Mut | OR  Name | №  Mut | OR  Name | №  Mut | OR  Name | №  Mut | OR  Name | №  Mut | OR  Name | №  Mut | OR  Name | №  Mut | OR  Name | №  Mut | OR  Name | №  Mut | OR  Name | №  Mut | OR  Name | №  Mut |
| **OR1A1** | 292 | **OR2A1** | 90 | **OR3A1** | 328 | **OR4A5** | 624 | **OR5A1** | 350 | **OR6A2** | 348 | **OR7A5** | 286 | **OR8A1** | 315 | **OR9A2** | 266 | **OR10A2** | 309 | **OR11A1** | 296 | **OR12D2** | 281 | **OR13A1** | 450 | **OR14A2** | 183 | **OR51A2** | 216 | OR52A1 | 310 | **OR56A1** | 329 |
| **OR1A2** | 241 | **OR2A2** | 312 | **OR3A2** | 329 | **OR4A15** | 566 | **OR5A2** | 312 | **OR6B1** | 293 | **OR7A10** | 306 | **OR8B2** | 295 | **OR9A4** | 239 | **OR10A3** | 326 | **OR11G2** | 314 | **OR12D3** | 259 | **OR13C2** | 359 | **OR14A16** | 256 | **OR51A4** | 295 | OR52A5 | 306 | **OR56A3** | 300 |
| **OR1B1** | 311 | **OR2A4** | 197 | **OR3A3** | 253 | **OR4A16** | 552 | **OR5AC2** | 341 | **OR6B2** | 349 | **OR7A17** | 345 | **OR8B3** | 297 | **OR9G1** | 246 | **OR10A4** | 332 | **OR11H1** | 256 |  |  | **OR13C3** | 336 | **OR14C36** | 288 | **OR51A7** | 282 | OR52B2 | 335 | **OR56A4** | 47 |
| **OR1C1** | 334 | **OR2A5** | 323 |  |  | **OR4A47** | 453 | **OR5AK2** | 371 | **OR6B3** | 322 | **OR7C1** | 284 | **OR8B4** | 261 | **OR9G4** | 353 | **OR10A5** | 338 | **OR11H4** | 354 |  |  | **OR13C4** | 272 | **OR14I1** | 314 | **OR51B2** | 328 | OR52B4 | 387 | **OR56B1** | 363 |
| **OR1D2** | 278 | **OR2A7** | 158 |  |  | **OR4B1** | 391 | **OR5AN1** | 324 | **OR6C1** | 310 | **OR7C2** | 278 | **OR8B8** | 314 | **OR9I1** | 291 | **OR10A6** | 382 | **OR11H6** | 320 |  |  | **OR13C5** | 363 | **OR14J1** | 296 | **OR51B4** | 327 | OR52B6 | 297 | **OR56B4** | 350 |
| **OR1D5** | 244 | **OR2A12** | 304 |  |  | **OR4C3** | 29 | **OR5AP2** | 293 | **OR6C2** | 315 | **OR7D2** | 299 | **OR8B12** | 284 | **OR9K2** | 359 | **OR10A7** | 299 | **OR11H12** | 331 |  |  | **OR13C8** | 274 | **OR14K1** | 255 | **OR51B5** | 412 | OR52D1 | 358 |  |  |
| **OR1E1** | 257 | **OR2A14** | 333 |  |  | **OR4C5** | 229 | **OR5AR1** | 336 | **OR6C3** | 352 | **OR7D4** | 339 | **OR8D1** | 291 | **OR9Q1** | 307 | **OR10C1** | 306 | **OR11L1** | 316 |  |  | **OR13C9** | 282 |  |  | **OR51B6** | 357 | OR52E2 | 308 |  |  |
| **OR1E2** | 296 | **OR2A25** | 318 |  |  | **OR4C6** | 425 | **OR5AS1** | 338 | **OR6C4** | 356 | **OR7E24** | 291 | **OR8D2** | 261 | **OR9Q2** | 358 | **OR10D3** | 239 |  |  |  |  | **OR13D1** | 324 |  |  | **OR51D1** | 381 | OR52E4 | 329 |  |  |
| **OR1F1** | 389 | **OR2A42** | 141 |  |  | **OR4C11** | 355 | **OR5AU1** | 405 | **OR6C6** | 300 | **OR7G1** | 319 | **OR8D4** | 296 |  |  | **OR10G2** | 381 |  |  |  |  | **OR13F1** | 325 |  |  | **OR51E1** | 39 | OR52E6 | 361 |  |  |
| **OR1G1** | 295 | **OR2B2** | 267 |  |  | **OR4C12** | 224 | **OR5B12** | 313 | **OR6C65** | 280 | **OR7G2** | 12 | **OR8G1** | 339 |  |  | **OR10G3** | 295 |  |  |  |  | **OR13G1** | 286 |  |  | **OR51E2** | 392 | OR52E8 | 372 |  |  |
| **OR1I1** | 372 | **OR2B3** | 266 |  |  | **OR4C13** | 323 | **OR5B17** | 301 | **OR6C68** | 336 | **OR7G3** | 284 | **OR8G5** | 332 |  |  | **OR10G4** | 324 |  |  |  |  | **OR13H1** | 210 |  |  | **OR51F1** | 311 | OR52H1 | 312 |  |  |
| **OR1J1** | 325 | **OR2B6** | 238 |  |  | **OR4C15** | 33 | **OR5B2** | 332 | **OR6C70** | 309 |  |  | **OR8H1** | 344 |  |  | **OR10G6** | 100 |  |  |  |  | **OR13J1** | 350 |  |  | **OR51F2** | 354 | OR52I1 | 348 |  |  |
| **OR1J2** | 295 | **OR2B11** | 334 |  |  | **OR4C16** | 514 | **OR5B21** | 293 | **OR6C74** | 279 |  |  | **OR8H2** | 328 |  |  | **OR10G7** | 362 |  |  |  |  |  |  |  |  | **OR51G1** | 375 | OR52I2 | 345 |  |  |
| **OR1J4** | 259 | **OR2C1** | 386 |  |  | **OR4C46** | 557 | **OR5B3** | 314 | **OR6C75** | 257 |  |  | **OR8H3** | 343 |  |  | **OR10G8** | 352 |  |  |  |  |  |  |  |  | **OR51G2** | 364 | OR52J3 | 337 |  |  |
| **OR1K1** | 346 | **OR2C3** | 342 |  |  | **OR4D1** | 267 | **OR5C1** | 352 | **OR6C76** | 285 |  |  | **OR8I2** | 345 |  |  | **OR10G9** | 372 |  |  |  |  |  |  |  |  | **OR51H1** | 284 | OR52K1 | 412 |  |  |
| **OR1L1** | 51 | **OR2D2** | 369 |  |  | **OR4D2** | 279 | **OR5D13** | 339 | **OR6F1** | 296 |  |  | **OR8J1** | 307 |  |  | **OR10H1** | 390 |  |  |  |  |  |  |  |  | **OR51I1** | 350 | OR52K2 | 408 |  |  |
| **OR1L3** | 343 | **OR2D3** | 342 |  |  | **OR4D5** | 324 | **OR5D14** | 344 | **OR6J1** | 240 |  |  | **OR8J3** | 315 |  |  | **OR10H2** | 361 |  |  |  |  |  |  |  |  | **OR51I2** | 384 | OR52L1 | 338 |  |  |
| **OR1L4** | 280 | **OR2F1** | 310 |  |  | **OR4D6** | 315 | **OR5D16** | 350 | **OR6K2** | 372 |  |  | **OR8K1** | 323 |  |  | **OR10H3** | 295 |  |  |  |  |  |  |  |  | **OR51J1** | 282 | OR52M1 | 485 |  |  |
| **OR1L6** | 47 | **OR2F2** | 340 |  |  | **OR4D9** | 333 | **OR5D18** | 294 | **OR6K3** | 31 |  |  | **OR8K3** | 321 |  |  | **OR10H4** | 304 |  |  |  |  |  |  |  |  | **OR51L1** | 279 | OR52N1 | 290 |  |  |
| **OR1L8** | 272 | **OR2G2** | 315 |  |  | **OR4D10** | 316 | **OR5F1** | 357 | **OR6K6** | 346 |  |  | **OR8K5** | 295 |  |  | **OR10H5** | 404 |  |  |  |  |  |  |  |  | **OR51M1** | 394 | OR52N2 | 335 |  |  |
| **OR1M1** | 379 | **OR2G3** | 275 |  |  | **OR4D11** | 307 | **OR5H1** | 355 | **OR6M1** | 312 |  |  | **OR8S1** | 374 |  |  | **OR10J1** | 407 |  |  |  |  |  |  |  |  | **OR51Q1** | 413 | OR52N4 | 341 |  |  |
| **OR1N1** | 291 | **OR2G6** | 412 |  |  | **OR4E2** | 306 | **OR5H14** | 404 | **OR6N1** | 319 |  |  | **OR8U1** | 208 |  |  | **OR10J3** | 331 |  |  |  |  |  |  |  |  | **OR51S1** | 368 | OR52N5 | 284 |  |  |
| **OR1N2** | 320 | **OR2H1** | 308 |  |  | **OR4F4** | 179 | **OR5H15** | 375 | **OR6N2** | 274 |  |  |  |  |  |  | **OR10J5** | 291 |  |  |  |  |  |  |  |  | **OR51T1** | 23 | OR52R1 | 315 |  |  |
| **OR1Q1** | 295 | **OR2H2** | 305 |  |  | **OR4F5** | 132 | **OR5H2** | 292 | **OR6P1** | 280 |  |  |  |  |  |  | **OR10K1** | 337 |  |  |  |  |  |  |  |  | **OR51V1** | 371 | OR52W1 | 324 |  |  |
| **OR1S1** | 363 | **OR2J1** | 275 |  |  | **OR4F6** | 320 | **OR5H6** | 430 | **OR6Q1** | 301 |  |  |  |  |  |  | **OR10K2** | 303 |  |  |  |  |  |  |  |  |  |  |  |  |  |  |
| **OR1S2** | 345 | **OR2J2** | 298 |  |  | **OR4F15** | 308 | **OR5I1** | 320 | **OR6S1** | 344 |  |  |  |  |  |  | **OR10P1** | 343 |  |  |  |  |  |  |  |  |  |  |  |  |  |  |
|  |  | **OR2J3** | 260 |  |  | **OR4F17** | 58 | **OR5J2** | 328 | **OR6T1** | 358 |  |  |  |  |  |  | **OR10Q1** | 389 |  |  |  |  |  |  |  |  |  |  |  |  |  |  |
|  |  | **OR2K2** | 29 |  |  | **OR4F21** | 56 | **OR5K1** | 352 | **OR6V1** | 267 |  |  |  |  |  |  | **OR10R2** | 327 |  |  |  |  |  |  |  |  |  |  |  |  |  |  |
|  |  | **OR2L2** | 366 |  |  | **OR4K1** | 392 | **OR5K2** | 333 | **OR6X1** | 283 |  |  |  |  |  |  | **OR10S1** | 363 |  |  |  |  |  |  |  |  |  |  |  |  |  |  |
|  |  | **OR2L3** | 315 |  |  | **OR4K2** | 382 | **OR5K3** | 331 | **OR6Y1** | 332 |  |  |  |  |  |  | **OR10T2** | 319 |  |  |  |  |  |  |  |  |  |  |  |  |  |  |
|  |  | **OR2L5** | 342 |  |  | **OR4K5** | 365 | **OR5K4** | 316 |  |  |  |  |  |  |  |  | **OR10V1** | 305 |  |  |  |  |  |  |  |  |  |  |  |  |  |  |
|  |  | **OR2L8** | 312 |  |  | **OR4K13** | 309 | **OR5L1** | 371 |  |  |  |  |  |  |  |  | **OR10W1** | 332 |  |  |  |  |  |  |  |  |  |  |  |  |  |  |
|  |  | **OR2L13** | 325 |  |  | **OR4K14** | 283 | **OR5L2** | 351 |  |  |  |  |  |  |  |  | **OR10X1** | 348 |  |  |  |  |  |  |  |  |  |  |  |  |  |  |
|  |  | **OR2M2** | 349 |  |  | **OR4K15** | 404 | **OR5M1** | 345 |  |  |  |  |  |  |  |  | **OR10Z1** | 339 |  |  |  |  |  |  |  |  |  |  |  |  |  |  |
|  |  | **OR2M3** | 345 |  |  | **OR4K17** | 21 | **OR5M10** | 381 |  |  |  |  |  |  |  |  | **OR10AD1** | 337 |  |  |  |  |  |  |  |  |  |  |  |  |  |  |
|  |  | **OR2M4** | 275 |  |  | **OR4L1** | 345 | **OR5M11** | 334 |  |  |  |  |  |  |  |  | **OR10AG1** | 316 |  |  |  |  |  |  |  |  |  |  |  |  |  |  |
|  |  | **OR2M5** | 384 |  |  | **OR4M1** | 413 | **OR5M3** | 349 |  |  |  |  |  |  |  |  |  |  |  |  |  |  |  |  |  |  |  |  |  |  |  |  |
|  |  | **OR2M7** | 315 |  |  | **OR4M2** | 362 | **OR5M8** | 372 |  |  |  |  |  |  |  |  |  |  |  |  |  |  |  |  |  |  |  |  |  |  |  |  |
|  |  | **OR2S2** | 323 |  |  | **OR4N2** | 378 | **OR5M9** | 347 |  |  |  |  |  |  |  |  |  |  |  |  |  |  |  |  |  |  |  |  |  |  |  |  |
|  |  | **OR2T1** | 386 |  |  | **OR4N4** | 378 | **OR5P2** | 400 |  |  |  |  |  |  |  |  |  |  |  |  |  |  |  |  |  |  |  |  |  |  |  |  |
|  |  | **OR2T2** | 400 |  |  | **OR4N5** | 322 | **OR5P3** | 315 |  |  |  |  |  |  |  |  |  |  |  |  |  |  |  |  |  |  |  |  |  |  |  |  |
|  |  | **OR2T3** | 351 |  |  | **OR4P4** | 275 | **OR5R1** | 319 |  |  |  |  |  |  |  |  |  |  |  |  |  |  |  |  |  |  |  |  |  |  |  |  |
|  |  | **OR2T4** | 367 |  |  | **OR4Q3** | 412 | **OR5T1** | 369 |  |  |  |  |  |  |  |  |  |  |  |  |  |  |  |  |  |  |  |  |  |  |  |  |
|  |  | **OR2T5** | 101 |  |  | **OR4S1** | 379 | **OR5T2** | 416 |  |  |  |  |  |  |  |  |  |  |  |  |  |  |  |  |  |  |  |  |  |  |  |  |
|  |  | **OR2T6** | 322 |  |  | **OR4S2** | 265 | **OR5T3** | 315 |  |  |  |  |  |  |  |  |  |  |  |  |  |  |  |  |  |  |  |  |  |  |  |  |
|  |  | **OR2T7** | 451 |  |  | **OR4X1** | 394 | **OR5V1** | 282 |  |  |  |  |  |  |  |  |  |  |  |  |  |  |  |  |  |  |  |  |  |  |  |  |
|  |  | **OR2T8** | 309 |  |  | **OR4X2** | 470 | **OR5W2** | 315 |  |  |  |  |  |  |  |  |  |  |  |  |  |  |  |  |  |  |  |  |  |  |  |  |
|  |  | **OR2T10** | 284 |  |  |  |  |  |  |  |  |  |  |  |  |  |  |  |  |  |  |  |  |  |  |  |  |  |  |  |  |  |  |
|  |  | **OR2T11** | 381 |  |  |  |  |  |  |  |  |  |  |  |  |  |  |  |  |  |  |  |  |  |  |  |  |  |  |  |  |  |  |
|  |  | **OR2T12** | 382 |  |  |  |  |  |  |  |  |  |  |  |  |  |  |  |  |  |  |  |  |  |  |  |  |  |  |  |  |  |  |
|  |  | **OR2T27** | 425 |  |  |  |  |  |  |  |  |  |  |  |  |  |  |  |  |  |  |  |  |  |  |  |  |  |  |  |  |  |  |
|  |  | **OR2T29** | 106 |  |  |  |  |  |  |  |  |  |  |  |  |  |  |  |  |  |  |  |  |  |  |  |  |  |  |  |  |  |  |
|  |  | **OR2T33** | 390 |  |  |  |  |  |  |  |  |  |  |  |  |  |  |  |  |  |  |  |  |  |  |  |  |  |  |  |  |  |  |
|  |  | **OR2T34** | 350 |  |  |  |  |  |  |  |  |  |  |  |  |  |  |  |  |  |  |  |  |  |  |  |  |  |  |  |  |  |  |
|  |  | **OR2T35** | 257 |  |  |  |  |  |  |  |  |  |  |  |  |  |  |  |  |  |  |  |  |  |  |  |  |  |  |  |  |  |  |
|  |  | **OR2V1** | 286 |  |  |  |  |  |  |  |  |  |  |  |  |  |  |  |  |  |  |  |  |  |  |  |  |  |  |  |  |  |  |
|  |  | **OR2V2** | 297 |  |  |  |  |  |  |  |  |  |  |  |  |  |  |  |  |  |  |  |  |  |  |  |  |  |  |  |  |  |  |
|  |  | **OR2W1** | 263 |  |  |  |  |  |  |  |  |  |  |  |  |  |  |  |  |  |  |  |  |  |  |  |  |  |  |  |  |  |  |
|  |  | **OR2W3** | 382 |  |  |  |  |  |  |  |  |  |  |  |  |  |  |  |  |  |  |  |  |  |  |  |  |  |  |  |  |  |  |
|  |  | **OR2Y1** | 352 |  |  |  |  |  |  |  |  |  |  |  |  |  |  |  |  |  |  |  |  |  |  |  |  |  |  |  |  |  |  |
|  |  | **OR2Z1** | 276 |  |  |  |  |  |  |  |  |  |  |  |  |  |  |  |  |  |  |  |  |  |  |  |  |  |  |  |  |  |  |
|  |  | **OR2AE1** | 327 |  |  |  |  |  |  |  |  |  |  |  |  |  |  |  |  |  |  |  |  |  |  |  |  |  |  |  |  |  |  |
|  |  | **OR2AG1** | 337 |  |  |  |  |  |  |  |  |  |  |  |  |  |  |  |  |  |  |  |  |  |  |  |  |  |  |  |  |  |  |
|  |  | **OR2AG2** | 373 |  |  |  |  |  |  |  |  |  |  |  |  |  |  |  |  |  |  |  |  |  |  |  |  |  |  |  |  |  |  |
|  |  | **OR2AJ1** | 207 |  |  |  |  |  |  |  |  |  |  |  |  |  |  |  |  |  |  |  |  |  |  |  |  |  |  |  |  |  |  |
|  |  | **OR2AK2** | 277 |  |  |  |  |  |  |  |  |  |  |  |  |  |  |  |  |  |  |  |  |  |  |  |  |  |  |  |  |  |  |
|  |  | **OR2AP1** | 297 |  |  |  |  |  |  |  |  |  |  |  |  |  |  |  |  |  |  |  |  |  |  |  |  |  |  |  |  |  |  |
|  |  | **OR2AT4** | 294 |  |  |  |  |  |  |  |  |  |  |  |  |  |  |  |  |  |  |  |  |  |  |  |  |  |  |  |  |  |  |

**Table S3: Human ORs genes with functional annotation excluded from the study.** The table shows the gene names of ORs that were excluded for variant annotation due to insufficient data in GnomAD v2 or lack of correspondence between GnomAD transcripts and UniProt reference sequences.

| **Gene** | **HORDE annotation** | **GnomAD Entry (Ensembl gene ID)** | **GnomAD/**  **Ensembl annotation** | **GnomAD variants (protein-coding)** | **UniProt ID** | **Reason to exclude from hORMdb** |
| --- | --- | --- | --- | --- | --- | --- |
| OR1D4 | [Functional](https://genome.weizmann.ac.il/horde/card/index/symbol:OR1D4) | [ENSG00000255095](https://gnomad.broadinstitute.org/gene/ENSG00000255095) | [Pseudogene](https://grch37.ensembl.org/Homo_sapiens/Gene/Summary?g=ENSG00000255095;r=17:3143970-3144908) | No variants found | [P47884](https://www.uniprot.org/uniprot/P47884) | No coding sequence variants found in GnomAD |
| OR1E3 | [Pseudogene](https://genome.weizmann.ac.il/horde/card/index/symbol:OR1E3P) | [ENSG00000142163](https://gnomad.broadinstitute.org/gene/ENSG00000142163) | [Pseudogene](https://grch37.ensembl.org/Homo_sapiens/Gene/Summary?g=ENSG00000142163;r=17:3019717-3020660) | No variants found | [Q8WZA6](https://www.uniprot.org/uniprot/Q8WZA6) | No coding sequence variants found in GnomAD |
| OR1F12 | [Functional](https://genome.weizmann.ac.il/horde/card/index/symbol:OR1F12) | [ENSG00000220721](https://gnomad.broadinstitute.org/gene/ENSG00000220721) | [Pseudogene](https://grch37.ensembl.org/Homo_sapiens/Gene/Summary?g=ENSG00000220721;r=6:28041094-28042011;t=ENST00000402365) | No variants found | [Q8NHA8](https://www.uniprot.org/uniprot/Q8NHA8) | No coding sequence variants found in GnomAD |
| OR1P1 | [Pseudogene](https://genome.weizmann.ac.il/horde/card/index/symbol:OR1P1P) | [ENSG00000262085](https://gnomad.broadinstitute.org/gene/ENSG00000262085) | [Pseudogene](https://grch37.ensembl.org/Homo_sapiens/Gene/Summary?g=ENSG00000262085;r=17:3057183-3058176;t=ENST00000571766) | No variants found | [Q8NH06](https://www.uniprot.org/uniprot/Q8NH06) | No coding sequence variants found in GnomAD |
| OR2W5 | [Functional](https://genome.weizmann.ac.il/horde/card/index/symbol:OR2W5) | [ENSG00000203664](https://gnomad.broadinstitute.org/gene/ENSG00000203664) | [Pseudogene](https://grch37.ensembl.org/Homo_sapiens/Gene/Summary?g=ENSG00000203664;r=1:247654370-247655710) | No variants found | [A6NFC9](https://www.uniprot.org/uniprot/A6NFC9) | No coding sequence variants found in GnomAD |
| OR3A4 | [Functional](https://genome.weizmann.ac.il/horde/card/index/symbol:OR3A4) | [ENSG00000180068](https://gnomad.broadinstitute.org/gene/ENSG00000180068) | [Pseudogene](https://grch37.ensembl.org/Homo_sapiens/Gene/Summary?g=ENSG00000180068;r=17:3213539-3214740) | No variants found | [P47883](https://www.uniprot.org/uniprot/P47883) | No coding sequence variants found in GnomAD |
| OR4A8 | [Pseudogene](https://genome.weizmann.ac.il/horde/card/index/symbol:OR4A8P) | [ENSG00000225997](https://gnomad.broadinstitute.org/gene/ENSG00000225997) | [Polymorphic pseudogene](https://grch37.ensembl.org/Homo_sapiens/Gene/Summary?g=ENSG00000225997;r=11:51435459-51436403;t=ENST00000418689) | Yes | [P0C604](https://www.uniprot.org/uniprot/P0C604) | GnomAD transcript does not match with UniProt sequence |
| OR4C45 | [Functional](https://genome.weizmann.ac.il/horde/card/index/symbol:OR4C45) | No entry found | [Pseudogene](https://grch37.ensembl.org/Homo_sapiens/Gene/Summary?db=core;g=ENSG00000260811;r=HG281_PATCH:50054412-50055332;t=ENST00000568934) | No entry found | [A6NMZ5](https://www.uniprot.org/uniprot/A6NMZ5) | No entry found in GnomAD |
| OR4E1 | [Pseudogene](https://genome.weizmann.ac.il/horde/card/index/symbol:OR4E1P) | [ENSG00000180636](https://gnomad.broadinstitute.org/gene/ENSG00000180636) | [Pseudogene](https://grch37.ensembl.org/Homo_sapiens/Gene/Summary?g=ENSG00000180636;r=14:22138203-22139150) | No variants found | [P0C645](https://www.uniprot.org/uniprot/P0C645) | No coding sequence variants found in GnomAD |
| OR4F3 | [Functional](https://genome.weizmann.ac.il/horde/card/index/symbol:OR4F3) | [ENSG00000230178](https://gnomad.broadinstitute.org/gene/ENSG00000230178) | [Protein coding](https://grch37.ensembl.org/Homo_sapiens/Gene/Summary?g=ENSG00000230178;r=5:180794269-180795263;t=ENST00000456475) | No variants found | [P0C645](https://www.uniprot.org/uniprot/P0C645) | No coding sequence variants found in GnomAD |
| OR4F16 | [Functional](https://genome.weizmann.ac.il/horde/card/index/symbol:OR4F16) | [ENSG00000185097](https://gnomad.broadinstitute.org/gene/ENSG00000185097) | [Protein coding](https://grch37.ensembl.org/Homo_sapiens/Gene/Summary?db=core;g=ENSG00000185097;r=1:621059-622053;t=ENST00000332831) | No variants found | [P0C646](https://www.uniprot.org/uniprot/P0C645) | No coding sequence variants found in GnomAD |
| OR4F29 | [Functional](https://genome.weizmann.ac.il/horde/card/index/symbol:OR4F29) | [ENSG00000235249](https://gnomad.broadinstitute.org/gene/ENSG00000235249) | [Protein coding](https://grch37.ensembl.org/Homo_sapiens/Gene/Summary?db=core;g=ENSG00000235249;r=1:367640-368634;t=ENST00000426406) | No variants found | [P0C647](https://www.uniprot.org/uniprot/P0C645) | No coding sequence variants found in GnomAD |
| OR4K3 | [Pseudogene](https://genome.weizmann.ac.il/horde/card/index/symbol:OR4K3P) | [ENSG00000176290](https://gnomad.broadinstitute.org/gene/ENSG00000176290) | [Pseudogene](https://grch37.ensembl.org/Homo_sapiens/Gene/Summary?g=ENSG00000176290;r=14:20336401-20337302) | No variants found | [Q96R72](https://www.uniprot.org/uniprot/Q96R72) | No coding sequence variants found in GnomAD |
| OR4Q2 | [Pseudogene](https://genome.weizmann.ac.il/horde/card/index/symbol:OR4Q2P) | [ENSG00000196383](https://gnomad.broadinstitute.org/gene/ENSG00000196383) | [Polymorphic pseudogene](https://grch37.ensembl.org/Homo_sapiens/Gene/Summary?g=ENSG00000196383;r=14:20470322-20471265;t=ENST00000541415) | Yes | [P0C623](https://www.uniprot.org/uniprot/P0C623) | GnomAD transcript does not match with UniProt sequence |
| OR5G3 | [Pseudogene](https://genome.weizmann.ac.il/horde/card/index/symbol:OR5G3P) | [ENSG00000241356](https://gnomad.broadinstitute.org/gene/ENSG00000241356) | [Polymorphic pseudogene](https://grch37.ensembl.org/Homo_sapiens/Gene/Summary?g=ENSG00000241356;r=11:56587052-56587980;t=ENST00000435756) | Yes | [P0C626](https://www.uniprot.org/uniprot/P0C626) | GnomAD transcript does not match with UniProt sequence |
| OR5H8 | [Pseudogene](https://genome.weizmann.ac.il/horde/card/index/symbol:OR5H8P) | [ENSG00000232535](https://gnomad.broadinstitute.org/gene/ENSG00000232535) | [Polymorphic pseudogene](https://grch37.ensembl.org/Homo_sapiens/Gene/Summary?g=ENSG00000232535;r=3:98030757-98031687;t=ENST00000394191) | Yes | [P0DN80](https://www.uniprot.org/uniprot/P0DN80) | GnomAD transcript does not match with UniProt sequence |
| OR5AC1 | [Pseudogene](https://genome.weizmann.ac.il/horde/card/index/symbol:OR5AC1P) | [ENSG00000213439](https://gnomad.broadinstitute.org/gene/ENSG00000213439) | [Polymorphic pseudogene](https://grch37.ensembl.org/Homo_sapiens/Gene/Summary?g=ENSG00000213439;r=3:97783316-97784240;t=ENST00000429239) | Yes | [P0C628](https://www.uniprot.org/uniprot/P0C628) | GnomAD transcript does not match with UniProt sequence |
| OR5AL1 | [Pseudogene](https://genome.weizmann.ac.il/horde/card/index/symbol:OR5AL1P) | [ENSG00000272987](https://gnomad.broadinstitute.org/gene/ENSG00000272987) | [Polymorphic pseudogene](https://grch37.ensembl.org/Homo_sapiens/Gene/Summary?g=ENSG00000272987;r=11:56180172-56181156;t=ENST00000440231) | Yes | [P0C617](https://www.uniprot.org/uniprot/P0C617) | GnomAD transcript does not match with UniProt sequence |
| OR8J2 | [Pseudogene](https://genome.weizmann.ac.il/horde/card/index/symbol:OR8J2P) | [ENSG00000254658](https://gnomad.broadinstitute.org/gene/ENSG00000254658) | [Polymorphic pseudogene](https://grch37.ensembl.org/Homo_sapiens/Gene/Summary?g=ENSG00000254658;r=11:55978334-55979298;t=ENST00000533152) | Yes | [Q8NGG1](https://www.uniprot.org/uniprot/Q8NGG1) | GnomAD transcript does not match with UniProt sequence |
| OR8U8 | [Functional](https://genome.weizmann.ac.il/horde/card/index/symbol:OR8U8) | No entry found | [Pseudogene](https://grch37.ensembl.org/Homo_sapiens/Gene/Summary?g=) | No entry found | [P0C7N1](https://www.uniprot.org/uniprot/P0C7N1) | No entry found in GnomAD |
| OR8U9 | [Functional](https://genome.weizmann.ac.il/horde/card/index/symbol:OR8U9) | No entry found | [Protein coding](https://grch37.ensembl.org/Homo_sapiens/Gene/Summary?db=core;g=ENSG00000262315;r=HG142_HG150_NOVEL_TEST:56143101-56152270) | No entry found | [P0C7N5](https://www.uniprot.org/uniprot/P0C7N5) | No entry found in GnomAD |
| OR9G9 | [Functional](https://genome.weizmann.ac.il/horde/card/index/symbol:OR9G9) | No entry found | [Protein coding](https://grch37.ensembl.org/Homo_sapiens/Gene/Summary?db=core;g=ENSG00000262191;r=HG151_NOVEL_TEST:56478942-56479859;t=ENST00000571130) | No entry found | [P0C7N8](https://www.uniprot.org/uniprot/P0C7N8) | No entry found in GnomAD |
| OR10J4 | [Pseudogene](https://genome.weizmann.ac.il/horde/card/index/symbol:OR10J4P) | [ENSG00000249730](https://gnomad.broadinstitute.org/gene/ENSG00000249730) | [Polymorphic pseudogene](https://grch37.ensembl.org/Homo_sapiens/Gene/Summary?g=ENSG00000249730;r=1:159401994-159402928;t=ENST00000504970) | Yes | [P0C629](https://www.uniprot.org/uniprot/P0C629) | GnomAD transcript does not match with UniProt sequence |
| OR10AC1 | [Pseudogene](https://genome.weizmann.ac.il/horde/card/index/symbol:OR10AC1P) | [ENSG00000176510](https://gnomad.broadinstitute.org/gene/ENSG00000176510) | [Polymorphic pseudogene](https://grch37.ensembl.org/Homo_sapiens/Gene/Summary?g=ENSG00000176510;r=7:143208026-143209004;t=ENST00000439431) | Yes | [Q8NH08](https://www.uniprot.org/uniprot/Q8NH08) | GnomAD transcript does not match with UniProt sequence |
| OR11H2 | [Functional](https://genome.weizmann.ac.il/horde/card/index/symbol:OR11H2) | [ENSG00000258453](https://gnomad.broadinstitute.org/gene/ENSG00000258453) | [Pseudogene](https://grch37.ensembl.org/Homo_sapiens/Gene/Summary?g=ENSG00000258453;r=14:20181104-20182079;t=ENST00000556246) | No variants found | [Q8NH07](https://www.uniprot.org/uniprot/Q8NH07) | No coding sequence variants found in GnomAD |
| OR11H7 | [Pseudogene](https://genome.weizmann.ac.il/horde/card/index/symbol:OR11H7P) | [ENSG00000258806](https://gnomad.broadinstitute.org/gene/ENSG00000258806) | [Polymorphic pseudogene](https://grch37.ensembl.org/Homo_sapiens/Gene/Summary?g=ENSG00000258806;r=14:20697561-20698500;t=ENST00000553765) | Yes | [Q8NGC8](https://www.uniprot.org/uniprot/Q8NGC8) | GnomAD transcript does not match with UniProt sequence |
| OR12D1 | [Pseudogene](https://genome.weizmann.ac.il/horde/card/index/symbol:OR12D1P) | [ENSG00000251608](https://gnomad.broadinstitute.org/gene/ENSG00000251608) | [Polymorphic pseudogene](https://grch37.ensembl.org/Homo_sapiens/Gene/Summary?g=ENSG00000251608;r=6:29385057-29386003;t=ENST00000514827) | Yes | [P0DN82](https://www.uniprot.org/uniprot/P0DN82) | GnomAD transcript does not match with UniProt sequence |
| OR13C7 | [Pseudogene](https://genome.weizmann.ac.il/horde/card/index/symbol:OR13C7P) | [ENSG00000243641](https://gnomad.broadinstitute.org/gene/ENSG00000243641) | [Polymorphic pseudogene](https://grch37.ensembl.org/Homo_sapiens/Gene/Summary?g=ENSG00000243641;r=9:36002906-36003864;t=ENST00000424348) | Yes | [P0DN81](https://www.uniprot.org/uniprot/P0DN81) | GnomAD transcript does not match with UniProt sequence |
| OR52A4 | [Functional](https://genome.weizmann.ac.il/horde/card/index/symbol:OR52A4) | [ENSG00000205494](https://gnomad.broadinstitute.org/gene/ENSG00000205494) | [Pseudogene](https://grch37.ensembl.org/Homo_sapiens/Gene/Summary?g=ENSG00000205494;r=11:5141849-5145743) | No variants found | [A6NMU1](https://www.uniprot.org/uniprot/A6NMU1) | No coding sequence variants found in GnomAD |
| OR52E1 | [Pseudogene](https://genome.weizmann.ac.il/horde/card/index/symbol:OR52E1P) | [ENSG00000273085](https://gnomad.broadinstitute.org/gene/ENSG00000273085) | [Polymorphic pseudogene](https://grch37.ensembl.org/Homo_sapiens/Gene/Summary?g=ENSG00000273085;r=11:5090802-5091727;t=ENST00000445557) | Yes | [Q8NGJ3](https://www.uniprot.org/uniprot/Q8NGJ3) | GnomAD transcript does not match with UniProt sequence |
| OR52Z1 | [Pseudogene](https://genome.weizmann.ac.il/horde/card/index/symbol:OR52Z1P) | [ENSG00000176748](https://gnomad.broadinstitute.org/gene/ENSG00000176748) | [Pseudogene](https://grch37.ensembl.org/Homo_sapiens/Gene/Summary?g=ENSG00000176748;r=11:5198944-5199891;t=ENST00000510471) | No variants found | [P0C646](https://www.uniprot.org/uniprot/P0C646) | No coding sequence variants found in GnomAD |
| OR56A5 | [Functional](https://genome.weizmann.ac.il/horde/card/index/symbol:OR52Z1P) | [ENSG00000188691](https://gnomad.broadinstitute.org/gene/ENSG00000188691) | [Pseudogene](https://grch37.ensembl.org/Homo_sapiens/Gene/Summary?g=ENSG00000188691;r=11:5988407-5989724) | No variants found | [P0C7T3](https://www.uniprot.org/uniprot/P0C7T3) | No coding sequence variants found in GnomAD |
| OR52E5 | [Functional](https://genome.weizmann.ac.il/horde/card/index/symbol:OR52E5) | No entry found | [No entry found](https://grch37.ensembl.org/Homo_sapiens/Gene/Summary?db=core;g=) | No entry found | [Q8NH55](https://www.uniprot.org/uniprot/Q8NH55) | No entry found in GnomAD |

**Table S4: Conserved topological sites with functional implication in the GPCR activity.** The table shows the topological domain location, BW number, type and percentages of most conserved amino acids in 30 topological positions identified as important for the function of class A GPCRs according to several studies. Conservation values were obtained from the alignment in Additional file 3.

| **GPCR Domain** | **BW Position** | **Most cons. (%) hOR** | **Most cons. (%)**  **Class A GPCRs** | **Functional Role** | **REFERENCE** |
| --- | --- | --- | --- | --- | --- |
| **TM1** | 1.49 | G (88%) | G (67%) | Part of the "GN” conserved motif at human and mouse ORs | PMID:26044705 |
|  | 1.50 | N (99%) | N (98%) | A conserved asparagine residue occupies this position in ORs and Class A GPCRs. Involved in hydrogen bond network with D2.50 and N7.49 stabilizing the TM1, TM2 and TM7 domain region | PMID: 9115256 |
| **TM2** | 2.50 | D (81%) | D (92%) | Negatively charged (D/E) residues occupy this position in most Class A GPCRs. Participates in stabilizing hydrogen bond networks with TM1 and TM7 residues. Also involved in the coordination of ions in some receptors | PMID: 29395784 PMID: 31855179 |
|  | 2.53 | Y (43%) F (23%) L (20%) | V (30%) F (21%) M (13%) | In Class A GPCRs, this position contains >80% of bulky/aromatic residues. Maybe implicated in the first stage of activation pathway through TM2-TM7 | PMID: 24041646 |
|  | 2.59 | P (98%) | P (36%) F (21%) L (18%) | A conserved proline in this position induces a structural bulge in the TM2 in several class A GPCRs | PMID: 22435816 |
| **TM3** | 3.25 | C (98%) | C (86%) | Involved in a disulfide bond with ECL2 in >80% of Class A GPCRs | PMID: 21864311 |
|  | 3.39 | E (81%) D (13%) | S (71%) G (12%) | Identified as a hot-spot position that leads to substantially higher stability for several Class A GPCRs in the inactive state. Also associated with the coordination of ions in some receptors | PMID: 28644022 |
|  | 3.40 | C (37%) S (22%) | I (40%) V (21%) L (19%) | Part of the "transmission switch" in Class A GPCRs involved in activation | PMID: 22300046 |
|  | 3.49 | D (99%) | D (68%) E (23%) | Part of the "[D/E]RY" motif in Class A GPCRs involved in activation | PMID: 17192495 |
|  | 3.50 | R (89%) | R (97%) | Part of the "[D/E]RY" motif in Class A GPCRs involved in activation | PMID: 17192495 |
|  | 3.51 | Y (80%) | Y (72%) | Part of the "[D/E]RY" motif in Class A GPCRs involved in activation | PMID: 17192495 |
|  | 3.54 | I (88%) | I (54%) V (36%) | A bulky hydrophobic residue involved in interactions with G-proteins in several Class A GPCRs | PMID: 24016604 PMID: 25205354 |
| **TM4** | 4.50 | W (57%) Y (17%) | W (96%) | A conserved tryptophan residue occupies this position in most class A GPCRs. | PMID: 21921973 |
|  | 4.53 | G (71%) | S (45%) G (32%) | A conserved glycine in this position is crucial for cell surface trafficking of model ORs. | PMID: 31974307 |
| **TM5** | 5.50 | P (39%) D (14%) | P (77%) | Part of the "transmission switch" in Class A GPCRs involved in activation | PMID: 22300046 |
|  | 5.57 | S (98%) | C (39%) L (13%) | Involved in the Class A GPCR activation pathway in several receptors | PMID: 31855179 |
|  | 5.58 | Y (97%) | Y (75%) | Involved in the Class A GPCR activation pathway in several receptors | PMID: 31855179 |
|  | 5.65 | I (47%) V (43%) | I (46%) V (16%) A (15%) | Part of the hydrophobic "[I/L]xxL" motif at the intracellular end of TM5. Involved in interactions with G-proteins in several receptors. Mutations, particularly to polar amino acids, at this position in class A GPCRs inhibit G-protein coupling. | PMID: 23235263 |
| **TM6** | 6.30 | R (63%) K (12%) | E (35%) K (15%) R (14%) | Part of the "ionic lock" in Class A GPCRs involved in activation | PMID: 22300046 |
|  | 6.32 | K (92%) | K (44%) R (32%) | Involved in interactions with G-proteins in several Class A GPCRs | PMID: 23245528 |
|  | 6.33 | A (90%) | A (31%) V (19%) L (10%) | Involved in interactions with G-proteins in several Class A GPCRs | PMID: 23245528 |
|  | 6.37 | C (95%) | L (39%) V (22%) I (19%) | A highly conserved cysteine in human ORs. A bulky hydrophobic residue in this position is involved in the TM6 movement during Class A GPCRs activation | PMID: 29498889 |
|  | 6.44 | V (84%) | F (80%) | Part of the "transmission switch" in Class A GPCRs involved in activation | PMID: 22300046 |
|  | 6.48 | Y (69%) F (23%) | W (78%) F (9%) | Part of the WxP motif on TM6 in the majority of Class A GPCRs involved in activation | PMID: 22032986 PMID: 19375807 |
|  | 6.50 | P (33%) T (33%) A (33%) | P (98%) | Part of the WxP motif on TM6 in the majority of Class A GPCRs involved in activation | PMID: 22032986 PMID: 19375807 |
| **TM7** | 7.46 | P (95%) | S (60%) C (13%) A (13%) | A highly conserved proline in human ORs. Participates in an extended H-bond network important for receptor activation | PMID: 20395291 PMID: 20192770 |
|  | 7.49 | N (97%) | N (77%) | Part of the NP7.50xxY motif essential for forming the active conformation, also participates in forming the G protein–binding site. | PMID: 29925258 |
|  | 7.50 | P (97%) | P (96%) | Part of the NP7.50xxY motif essential for forming the active conformation, also participates in forming the G protein–binding site. | PMID: 29925258 |
|  | 7.53 | Y (96%) | Y (92%) | Part of the NP7.50xxY motif essential for forming the active conformation, also participates in forming the G protein–binding site. | PMID: 29925258 |
| **ECL2** | 45.50 | C (99%) | C (>80%) | Involved in a disulfide bond with the extracellular side of the TM3 in the majority of class A GPCRs | PMID: 21864311 |

**Table S5: Non-olfactory class A GPCRs used in topological annotation.** The table shows information of non-olfactory receptors with solved 3D-structures used in topological annotation and ligand binding cavity (BC) definition of OR natural variants (see Additional file 2: Fig. S5).

| **UniProtKB**  **entry name** | **Receptor Name** | | **Organism** | | **Resolution (Å)** | | **Ligand Name** | | **Ligand Function** | | **PDBid** |  |
| --- | --- | --- | --- | --- | --- | --- | --- | --- | --- | --- | --- | --- |
| **5HT2A** | | 5-Hydroxytryptamine receptor 2A | Human | | 2.9 | | Zotepine | | Antagonist | | 6A94 | |
| **5HT1B** | | 5-Hydroxytryptamine receptor 1B | Human | | 2.8 | | Dihydroergotamine | | Agonist | | 4IAQ | |
| **5HT2B** | | 5-Hydroxytryptamine receptor 2B | Human | | 2.7 | | Ergotamine | | Agonist | | 4IB4 | |
| **5HT2C** | | 5-Hydroxytryptamine receptor 2C | Human | | 2.7 | | Ritanserin | | Inverse agonist | | 6BQH | |
| **AA1R** | | Adenosine receptor A1 | Human | | 3.2 | | CHEMBL144360 | | Antagonist | | 5UEN | |
| **AA2AR** | | Adenosine receptor A2a | Human | | 2.7 | | ZM241385 | | Antagonist | | 3VG9 | |
| **ACM1** | | Muscarinic acetylcholine receptor M1 | Human | | 2.7 | | CHEMBL258622 | | Antagonist | | 5CXV | |
| **ACM2** | | Muscarinic acetylcholine receptor M2 | Human | | 2.3 | | N-methyl scopolamine | | Antagonist | | 5ZKC | |
| **ACM4** | | Muscarinic acetylcholine receptor M4 | Human | | 2.6 | | Tiotropium | | Antagonist | | 5DSG | |
| **ADRB1** | | Beta-1 adrenergic receptor | Turkey | | 2.3 | | (S)-Carvedilol | | Inverse agonist | | 4AMJ | |
| **ADRB2** | | Beta-2 adrenergic receptor | Human | | 3.2 | | Carazolol | | Inverse agonist | | 5JQH | |
| **AGTR1** | | Type-1 angiotensin II receptor | Human | | 2.8 | | OLM | | Inverse agonist | | 4ZUD | |
| **CNR1** | | Cannabinoid receptor 1 | Human | | 2.8 | | SCHEMBL662960 | | Antagonist | | 5TGZ | |
| **CNR2** | | Cannabinoid receptor 2 | Human | | 2.8 | | AM10257 | | Antagonist | | 5ZTY | |
| **CXCR4** | | C-X-C chemokine receptor type 4 | Human | | 2.5 | | IT1t | | Antagonist | | 3ODU | |
| **DRD2** | | Dopamine D2 receptor | Human | | 2.9 | | Risperidone | | Inverse agonist | | 6CM4 | |
| **DRD3** | | Dopamine D3 receptor | Human | | 2.9 | | Eticlopride | | Antagonist | | 3PBL | |
| **DRD4** | | Dopamine D4 receptor | Human | | 2.1 | | Nemonapride | | Antagonist | | 5WIV | |
| **EDNRB** | | Endothelin receptor type B | Human | | 2.2 | | K-8794 | | Antagonist | | 5X93 | |
| **HRH1** | | Histamine H1 receptor | Human | | 3.1 | | Doxepin | | Antagonist | | 3RZE | |
| **MTR1A** | | Melatonin receptor type 1A | Human | | 2.8 | | Ramelteon | | Agonist | | 6ME2 | |
| **NK1R** | | Tachykinin receptor 1 | Human | | 2.2 | | Netupitant | | Antagonist | | 6HLP | |
| **NPY1R** | | Neuropeptide Y receptor type 1 | Human | | 3.0 | | BMS-193885 | | Antagonist | | 5ZBH | |
| **OPRD** | | Delta-type opioid receptor | Human | | 1.8 | | Naltrindole | | Antagonist | | 4N6H | |
| **OPRK** | | Kappa-type opioid receptor | Human | | 2.9 | | JDTic | | Antagonist | | 4DJH | |
| **OPRM** | | Mu-type opioid receptor | Mouse | | 2.8 | | BF0 | | Antagonist | | 4DKL | |
| **OPRX** | | Nociceptin receptor | Human | | 3.0 | | N/A | | N/A | | 5DHG | |
| **OPSD** | | Rhodopsin | Bovine | | 2.7 | | Retinal | | Inverse agonist | | 1GZM | |
| **OX1R** | | Orexin receptor type 1 | Human | | 2.8 | | Suvorexant | | Antagonist | | 4ZJ8 | |
| **OX2R** | | Orexin receptor type 2 | Human | | 2.3 | | EMPA | | Antagonist | | 5WS3 | |
| **P2RY1** | | P2Y purinoceptor 1 | Human | | 2.7 | | MRS2500 | | Antagonist | | 4XNW | |
| **P2Y12** | | P2Y purinoceptor 12 | Human | | 2.6 | | AZD1283 | | Antagonist | | 4NTJ | |
| **PAR1** | | Proteinase-activated receptor 1 | Human | | 2.2 | | Vorapaxar | | Antagonist | | 3VW7 | |
| **PTAFR** | | Platelet-activating factor receptor | Human | | 2.9 | | ABT-491 | | Inverse agonist | | 5ZKQ | |
| **LPAR1** | | Lysophosphatidic acid receptor 1 | Human | | 2.9 | | ONO-9910539 | | Antagonist | | 4Z35 | |
| **S1PR1** | | Lysophospholipid (S1P) | Human | | 2.8 | | 909725-61-7 | | Antagonist | | 3V2Y | |
| **PE2R3** | | Prostanoid | Human | | 2.5 | | Misoprostol-FA | | Antagonist | | 6M9T | |
| **PE2R4** | | Prostanoid | Human | | 3.2 | | ONO-AE3-208 | | Antagonist | | 5YWY | |
| **TA2R** | | Prostanoid | Human | | 3.0 | | Daltroban | | Antagonist | | 6IIV | |
