# Supplementary material for: The mutational landscape of human olfactory G protein-coupled receptors
Source: BMC Biol. 2021 Feb 5;19:21. doi: 10.1186/s12915-021-00962-0 (PMC7866472; doi:10.1186/s12915-021-00962-0)
Supplement: Supplementary file 2 — Additional file 2: Figure S1. Chromosomal distribution of natural variants within OR families. Figure S2. Topological distribution of natural variants within OR families. Figure S3. The GPCRtm amino acid substitution scores. Figure S4. Functional core (FC) topological positions in class A GPCRs. Figure S5. Binding cavity (BC) topological positions in class A GPCRs. Figure S6. Human OR mutations with potential functional effects. Figure S7. Structure-based sequence alignment used in topological annotation. [file 12915_2021_962_MOESM2_ESM.docx]

**Additional file 2**

**The mutational landscape of human olfactory G protein-coupled receptors**

Ramón Cierco Jimenez ^1^, Nil Casajuana-Martin ^1^, Adrián García-Recio ^1^, Lidia Alcántara ^1^, Leonardo Pardo ^1^, Mercedes Campillo ^1^ and Angel Gonzalez ^1,^*

^1^ Laboratori de Medicina Computacional, Unitat de Bioestadística, Facultat de Medicina, Universitat Autònoma de Barcelona, E-08193 Bellaterra, Spain.

Present Address: Ramón Cierco Jiménez, International Agency for Research on Cancer, Evidence Synthesis and Classification Section, WHO Classification of Tumours Group, 150 Cours Albert Thomas, 69008 Lyon, France.

* To whom correspondence should be addressed.

Email: Angel.Gonzalez@uab.es

**Fig. S1: Chromosomal distribution of natural variants within OR families. A.** Total number of collected variants (y-axis) at each of the 17 OR families analyzed (x-axis). Bars are colored according to the chromosomal location of the natural variants (color legend on the right). **B.** Relative frequencies of the chromosomal distribution of the mutations at each OR family.

**
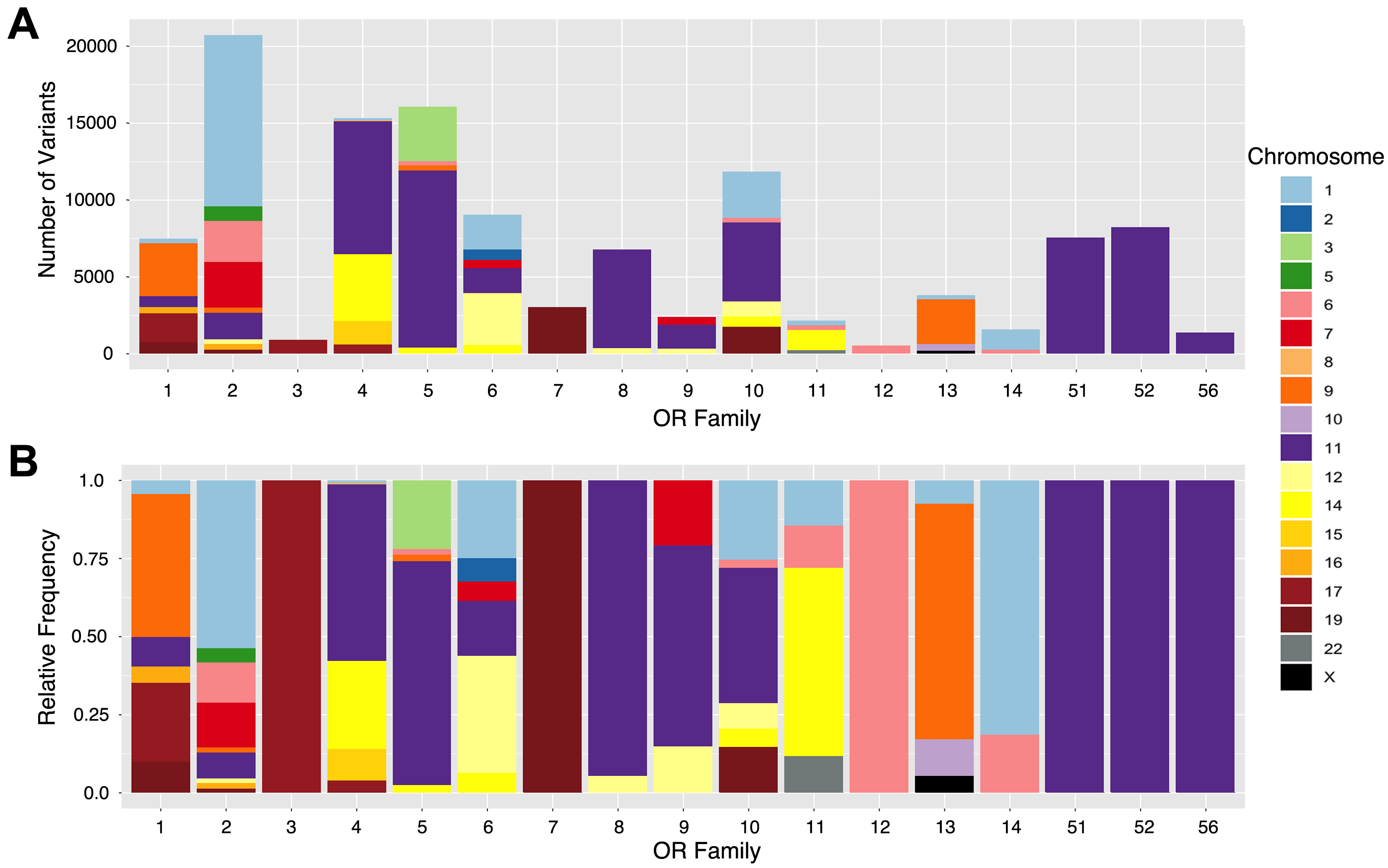
**

**Fig. S2: Topological distribution of natural variants within OR families. A.** Total number of collected variants (y-axis) at each of the 17 OR families analyzed (x-axis) color-coded by their GPCR domain location (color legend on the right). **B.** Relative frequencies of the topological domain distribution of the natural variants at each OR family.

**
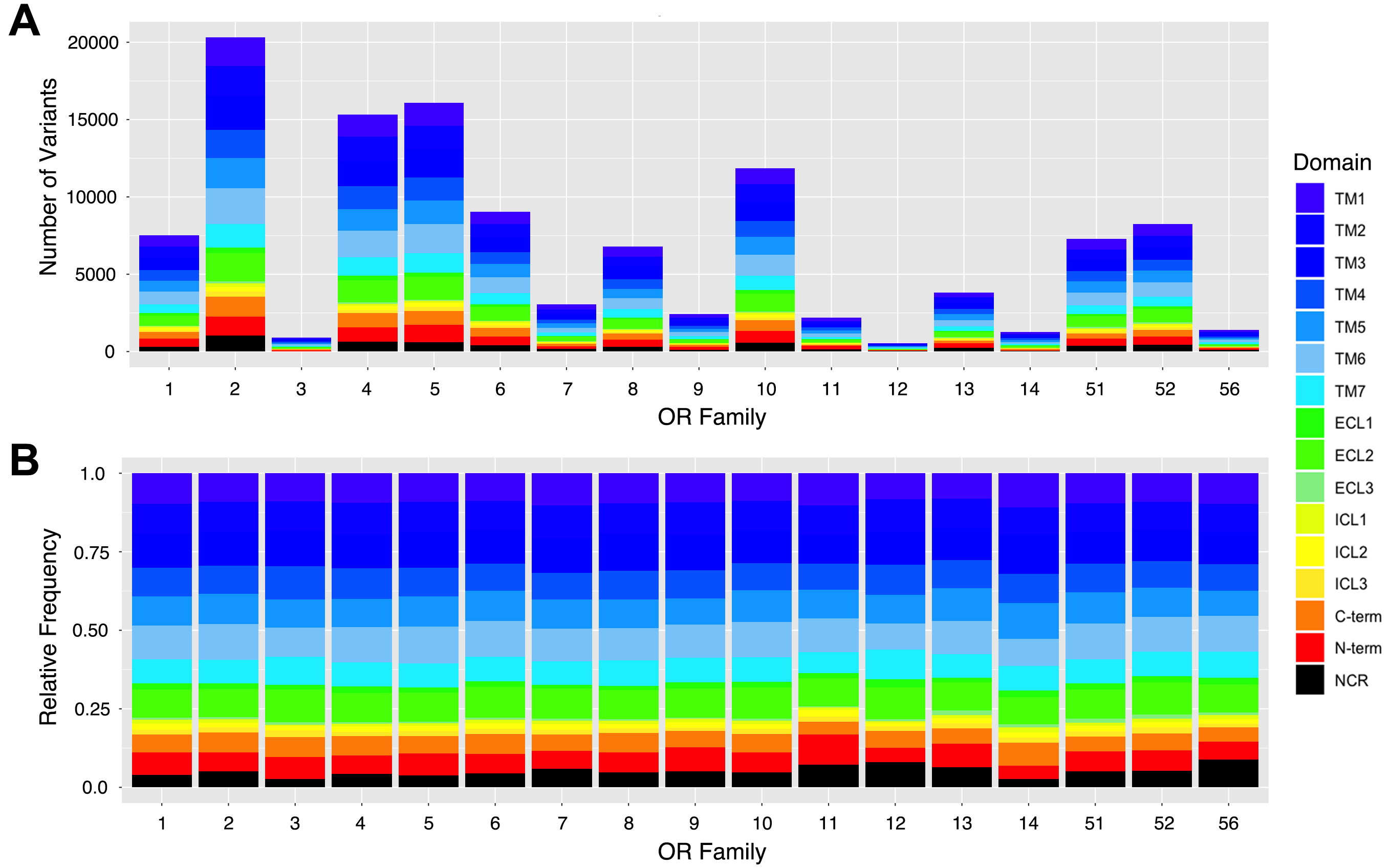
**

**Fig. S3: The GPCRtm amino acid substitution scores.** Values in the matrix correspond to statistical amino acid substitution scores calculated for the 20 aminoacids (one-letter code) in a MSA of more than one thousand class A GPCR sequences including human ORs.

**
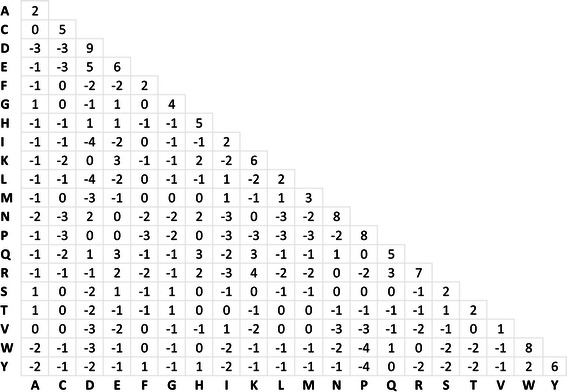
**

**Fig. S4: Functional core (FC) topological positions in class A GPCRs.** Snake plot representation of a generic class A GPCR with topological regions labeled. Color filled circles with BW notation indicate positions likely involved in the receptor activation or G-protein interaction (red) and conserved cysteines forming part of disulfide bridges (in yellow). Residue conservation and reference information to each position are available in the Additional file 1: Table S4.

**
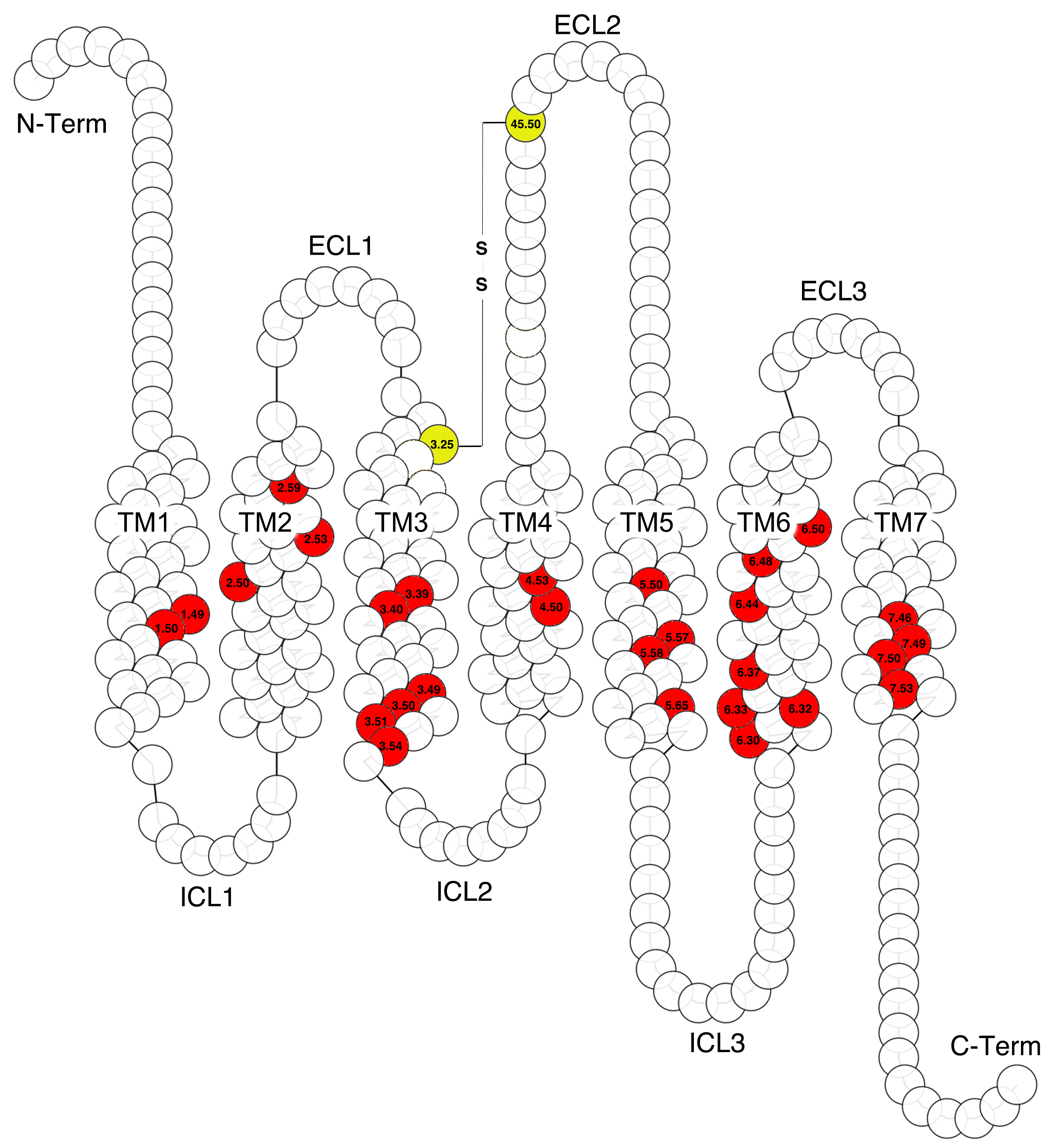
**

**Fig. S5: Binding cavity topological positions in class A GPCRs. A.** Structural superimposition of 39 class A GPCRs with known three-dimensional structures used for a general binding cavity (BC) definition (the complete list of the receptors is available in the Additional file1: Table S5). The molecular coordinates of the TM regions of each receptor with their corresponding ligands are represented in color tubes and sticks, respectively. **B.** Snake plot representation of the extracellular view of a generic GPCR with the TM regions indicated by numbers. Color filled circles with BW notation indicate the positions within a distance ≤ 4.0 Å of ligands in the crystallographic structures displayed in A.

**
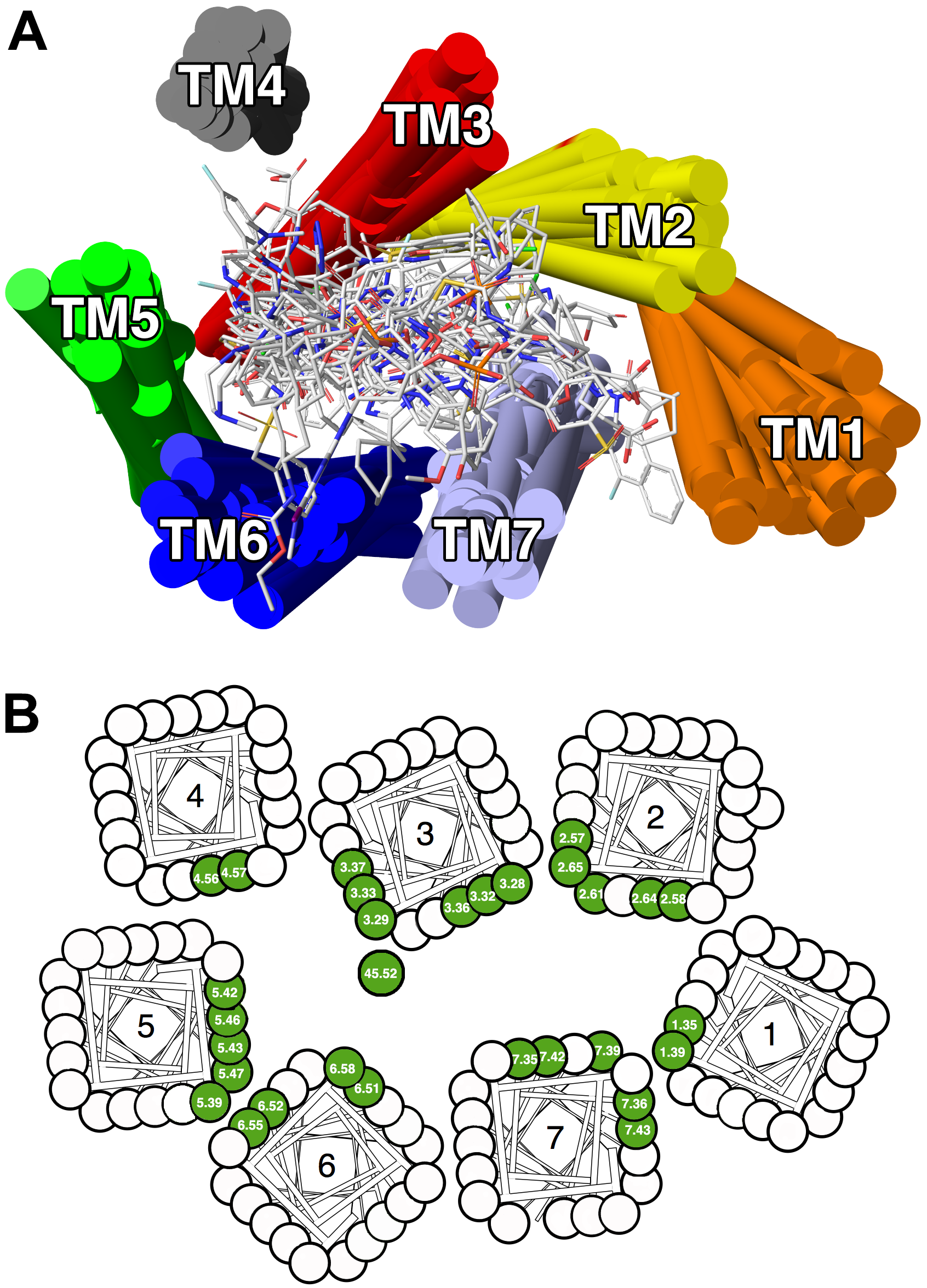
**

**Fig. S6: Human OR mutations with potential functional effects.** Eighty natural variants selected from the study with allele frequencies > 1% and belonging to FC and BC topological regions (see methods). For each natural variant a reference rsID number, BW position, type of substitution, functional region and gene ID are provided. Colored boxes indicate the allele frequencies expressed as a percentage (color legend on the right) in seven sub-continental populations: African (AFR), Ashkenazi Jewish (ASH), European Finnish (EF), European non-Finnish (ENF), Latino (LAT), East Asian (EA) and South Asian (SA).

**
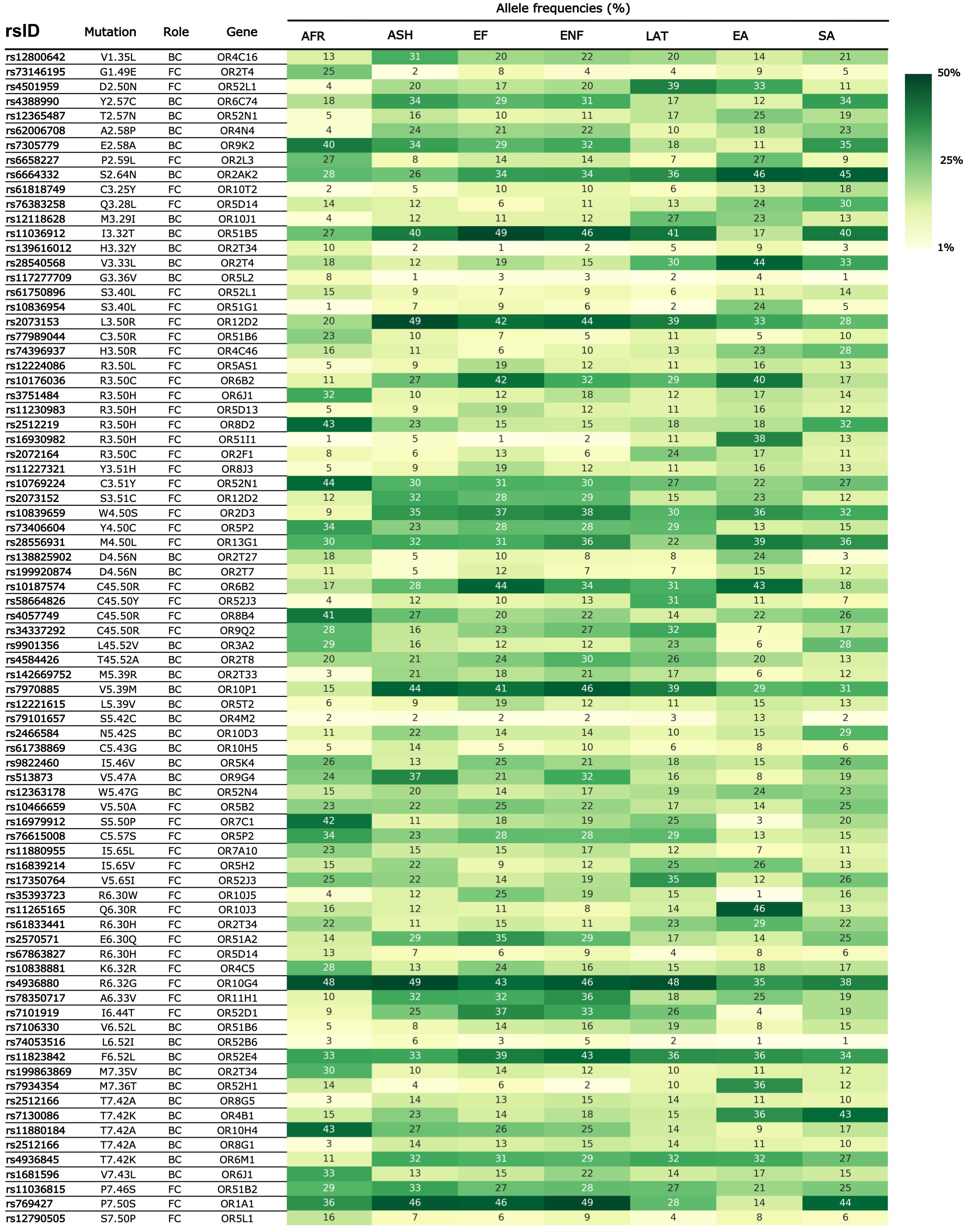
**

**Fig. S7: Structure-based sequence alignment used in topological annotation.** Sequence alignment of the TM regions of representative non-olfactory class A GPCRs with known 3D-structures and one member of each of the 17 OR families analyzed (the complete list of receptors is available in the Additional file 1). Non-olfactory receptors in the figure correspond to 5-hydroxytryptamine 2A (5HT2a, PDBid: 6A94), acetylcholine muscarinic (ACM1, PDBid: 6OIJ), 2-beta adrenergic (ADRB2, PDBid: 5JQH), dopamine D2 (DRD2, PDBid: 6CM4), histamine H1 (HRH1, PDBid: 3RZE), adenosine A2A (AA2AR, PDBid: 3VG9), rhodopsin (OPSD, PDBid: 1GZM), cannabinoid (CNR1, PDBid: 5TGZ) and Sphingosine 1-phosphate receptor (S1PR1, PDBid: 3V2Y). On the lower right is shown the structural superimposition of their TM regions with the most conserved BW positions (.50) at each helix highlighted. Ribbon diagrams on top of the alignment indicate the boundaries of the TM regions according to the structural superposition. The red frame in the alignment indicates BW positions. An adaptation of this numbering system was applied for a conserved stretch of 10 residues at the ECL2 (indicated by 45 as first number attending to its location between the TMs 4 and 5).

**
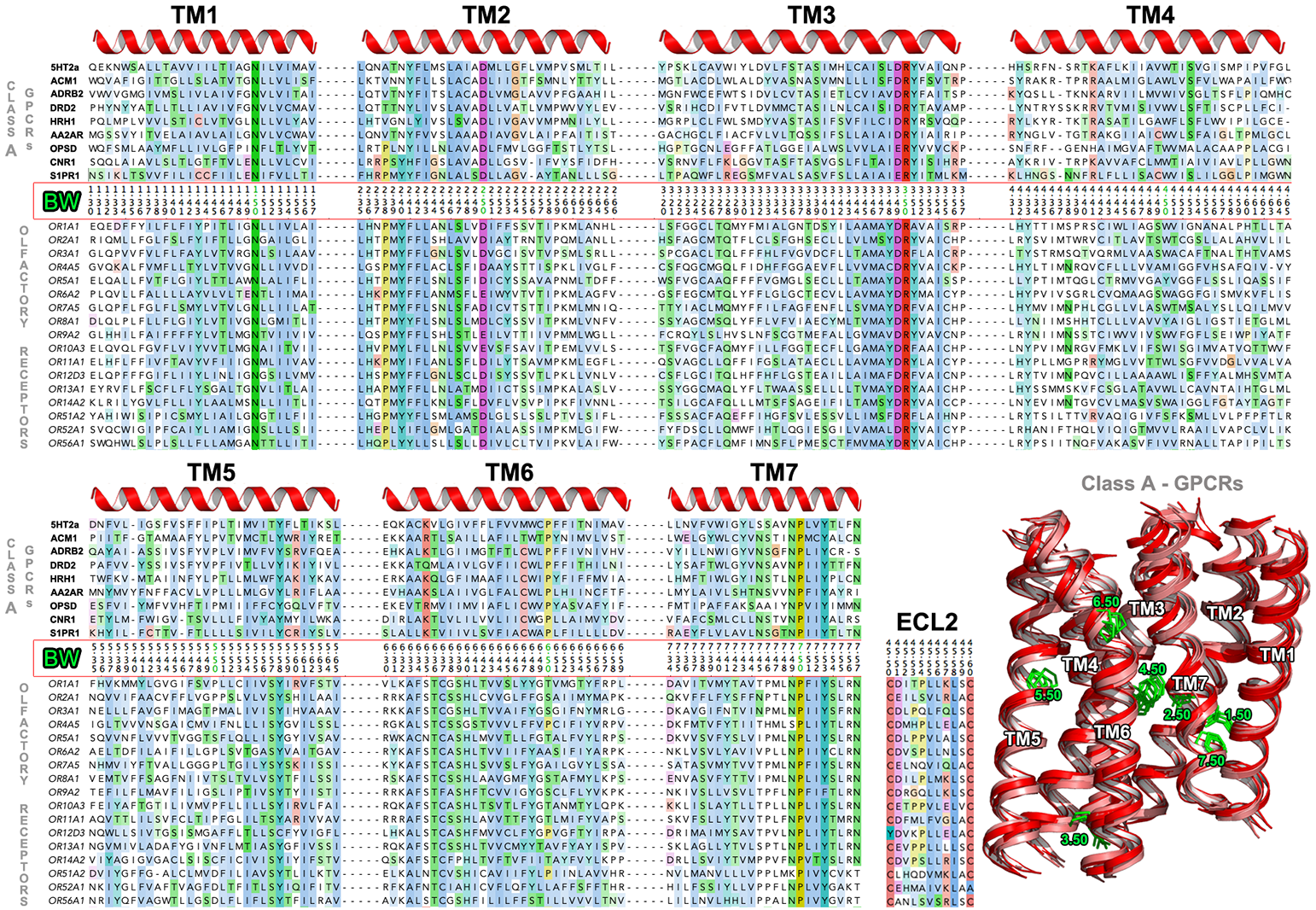
**
